# Incidence of unanticipated difficult airway using an objective airway score versus a standard clinical airway assessment: the DIFFICAIR trial – trial protocol for a cluster randomized clinical trial

**DOI:** 10.1186/1745-6215-14-347

**Published:** 2013-10-23

**Authors:** Anders Kehlet Nørskov, Charlotte Valentin Rosenstock, Jørn Wetterslev, Lars Hyldborg Lundstrøm

**Affiliations:** 1 Department of Anaesthesiology, Copenhagen University Hospital, Nordsjælland Hospital, 3400, Hillerød, Denmark; 2 Copenhagen Trial Unit, Centre for Clinical Intervention Research, Copenhagen University Hospital, Rigshospitalet 2100, Copenhagen, Denmark

**Keywords:** Airway management, Cluster analysis, Difficult intubation, Randomized controlled trials, Sensitivity, Specificity

## Abstract

**Background:**

Pre-operative airway assessment in Denmark is based on a non-specific clinical assessment. Systematic, evidence-based and consistent airway assessment may reduce the incidence of unanticipated difficult airway management. By assessing multiple predictors for difficult airway management, the predictive value of the assessment increases. The Simplified Airway Risk Index (SARI) is a multivariate risk score for predicting difficult intubation.

This study aims to compare the use of the SARI with a non-specified clinical airway assessment on predicting difficult intubation. Further, to compare the examination and registration of predictors for difficult mask ventilation with a non-specified clinical airway assessment on prediction of difficult mask ventilation.

**Method/Design:**

We cluster-randomized 28 Danish departments of anaesthesia to airway assessment either by the SARI or by usual non-specific assessment. Data from patients’ pre-operative airway assessment are registered in the Danish Anaesthesia Database. Objective scores for intubation and mask ventilation grade the severity of airway managements. The accuracy of predicting difficult intubation and mask ventilation is measured for each group. The primary outcome measure is the fraction of unanticipated difficult and easy intubation.

The fraction of unanticipated difficult intubation in Denmark is 1.87%. With a stratified randomization, type 1 error risk of 5% and a power of 80%, 30 departments are required to detect or reject a 30% relative risk reduction equalling a number needed to treat of 180. Sample size estimation is adjusted for the study design and based on standards for randomization on cluster-level. With an average cluster size of 2,500 patients, 70,000 patients will be enrolled over a 1-year trial period. The database is programmed so that registration of the SARI and predictors for difficult mask ventilation are mandatory for the intervention group but invisible to controls.

**Discussion:**

It is innovative to use a national clinical database as the basis for a randomized clinical trial. The method can serve as a precedent for implementation of evidence-based recommendations and database registration.

The trial will forward understanding of how to predict and reduce unanticipated difficult airways and how to produce evidence-based recommendations for airway assessment and clinical database development.

**Trial registration:**

(NCT01718561)

## Background

Unanticipated difficult airways are dreaded amongst anaesthesiologists and difficult tracheal intubation and difficult mask ventilation (DMV) can cause serious patient complications [1-4]. Better prediction of unanticipated difficult airways may reduce morbidity and mortality by allocating experienced personnel and relevant equipment. There is no single predictor that is sufficiently valid in predicting difficult tracheal intubation [5-10]. However, several studies show that by combining multiple predictors of difficult tracheal intubation, the positive and the negative predictive value of the assessment increases [10].

Mask ventilation is an essential component of airway management during general anaesthesia. In the event of failed intubation, establishing successful mask ventilation and thus oxygenation of the patient can be a life-saving procedure; DMV is correlated with difficult tracheal intubation [11,12]. A situation with both DMV and difficult tracheal intubation may place the patient at serious risk of complications or even death. Few studies have examined predictors for DMV and the frequency of the event [11,12]. There are no clear recommendations for when a patient should be considered at risk of DMV.

The American Society of Anesthesiologists (ASA) recommends a preoperative assessment of the patient’s airway based on 11 anatomical parameters [13,14]. Despite the ASA recommendation, there is no defined recommendation on which factors are mandatory for examination, nor on how these should be weighted in an overall airway assessment, and some of the critical cut-off values for the factors are not clearly defined. The ASA argues that the decision to assess some or all risk factors depends on the clinical context and it is left to the discretion of the individual anaesthesiologist [13,14]. In the UK, the Difficult Airway Society guidelines for management of the unanticipated difficult intubation [15] does not recommend preoperative airway assessment because of disputes about its value. However, the recently published Fourth National Audit Project [16] is opening up for a recommendation of a preoperative airway assessment although it has not been further defined.

The Danish Anaesthesia Database (DAD) is a clinical database that contains selected quantifiable indicators, covering the anaesthetic process from the preoperative assessment through anaesthesia and surgery to the post-operative recovery period. At present, all patient records in the database include the anaesthesiologist’s unspecified assessment of potential airway difficulties as well as a scheduled airway management plan. For all patients receiving general anaesthesia with an attempted intubation, an airway management score is registered based on the conditions of the (attempted) intubation. Likewise, in patients with attempted mask ventilation, an airway management score is registered for (attempted) mask ventilation.

In agreement with the ASA recommendations, the preoperative airway assessment in DAD is currently based exclusively on the individual anaesthesiologist’s preoperative clinical assessment, which is more or less based on various known, unknown, or less verified predictors of a difficult airway. Based on this assessment, whether mask ventilation and/or tracheal intubation by direct laryngoscopy is expected to be difficult is recorded as yes or no. Subsequently, the strategy for airway management is planned and recorded. There is little documentation of how accurately this preoperative clinical assessment predicts actual airway management conditions.

**Figure 1:**
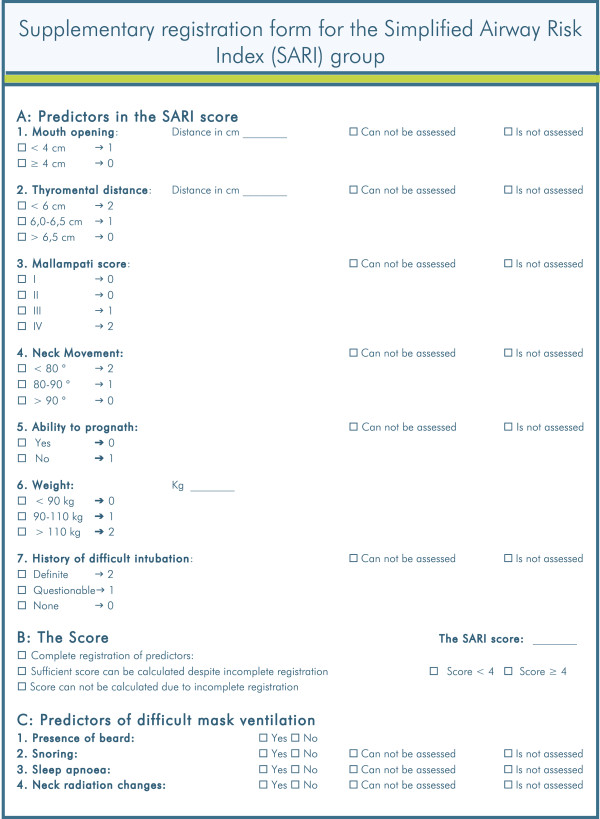
**Supplementary registration form for the SARI group.** This form, or a similar sticker, is attached to the anaesthesia record in the SARI group.

The Simplified Airway Risk Index (SARI) is a multivariate model for airway assessment described by El-Ganzouri et al., enabling an estimation of the likelihood of a difficult direct laryngoscopy [17]. The SARI contains seven individual predictors for difficult direct laryngoscopy, each given a weighted score 0–1 or 0–2. A summed value of the SARI score >3 indicates a future direct laryngoscopy to become difficult (Figure 1). It is unknown whether the SARI score predicts difficult intubation better or worse than a clinical assessment. We will compare the effect of using the SARI with an unspecified clinical airway assessment on the prediction of difficult intubation by direct laryngoscopy in a randomized clinical trial. Further, we want to record known risk factors for DMV and to investigate whether systematic registration of these risk factors leads to a reduction in DMV. During the DIFFICAIR trial, an internet page in the DAD will enable pre-operative registration of risk factors comprised in the SARI model. Kheterpal et al. described several risk factors associated with DMV [11,12]. Predictors for DMV will be a part of the data assessed and recorded in DAD in addition to the SARI score.

### Null hypothesis

• There is no difference in the proportion of unanticipated difficult intubations when the preoperative airway assessment is based on the SARI score compared with a preoperative airway assessment based on the individual anaesthesiologist’s assessment.

• There is no difference in the proportion of unanticipated DMV when the preoperative airway assessment includes systematic examination and registration of known predictors for DMV compared with an unstructured examination.

## Methods/Design

The trial is a cluster (cluster = department) and parallel group randomized trial stratified for the proportion of unanticipated difficult intubation. A total of 28 Danish departments of anaesthesia participate in the DIFFICAIR trial. They are randomized 1:1 as intervention departments with systematic airway assessment according to the SARI score and registration in the DAD or as control departments with preoperative airway assessment based on the individual anaesthesiologists’ assessment.

### Randomization

We conducted a baseline study in 2011 using data from the DAD version 3 and determined the proportion of unanticipated difficult intubation for each department of anaesthesia. The departments were then stratified according to whether the proportion of unanticipated difficult intubation was above or below 2%.

With appropriate use of allocation concealment, the heads of departments provided written informed consent before the departments were randomized. Thereafter, according to a computer-generated list of the allocation sequence, the departments were randomly assigned to one of two groups. In one group, anaesthesiologists are trained in preoperative use of the SARI score (the SARI group) and in a control group the preoperative airway assessments of the anaesthesiologists are based solely on a clinical assessment (CA group). The SARI group is thus included in a trial branch in which each patient has a preoperative airway assessment and a matching DAD registration consisting of a fixed panel of predetermined predictors for difficult intubation. In the SARI group four additional variables, which may be associated with DMV, are also recorded in DAD. Departments in the CA group continue to use an individual assessment of each patient regarding on whether the airway management will become difficult or not; this is preoperatively registered in DAD.

### Cluster randomization vs. individual randomization

Anaesthesiologists taught the use of the SARI score will inevitably and unintentionally use this knowledge during airway assessments also when assessing patients randomized for a “clinical assessment” only [18,19]. Therefore, a trial design using individual randomization of anaesthesiologists and patients is prone to yield incorrect results for the comparison of the two assessment methods. This is due to a “spill-over” effect between the trial branches within departments. Accordingly, we chose to randomize patients clustered on a departmental level [19].

### Inclusion

Departments registering patients in the DAD with an expected minimum of 200 intubations annually are eligible for inclusion.

Three populations of randomized patients are identified: Population 1: All patients primarily (attempted) intubated by direct laryngoscopy; Population 2: All patients primarily (attempted) intubated by direct laryngoscopy plus patients that are expected to be difficult to intubate by direct laryngoscopy and are therefore scheduled for intubation with an advanced method (e.g., video laryngoscopic or fibre-optic intubation); Population 3: All patients undergoing mask ventilation.

### Exclusion

Children <15 years old.

### Primary outcome measures

The following are measured regardless of randomization: i)Fraction of unanticipated difficult intubations = intubations with unanticipated difficulties [False negative]/all patients primarily (attempted) intubated by direct laryngoscopy; ii) Fraction of unanticipated easy intubations = intubations with anticipated difficulties that were easy [False Positive]/all patients primarily (attempted) intubated by direct laryngoscopy. Simultaneous low fractions of the primary outcome measures are desirable for good prediction of difficult intubation.

### Secondary outcome measures

• 48-hour mortality

• 30-day mortality

• Fraction = intubations anticipated to be difficult, thus planned for, and intubated by, an advanced method/all patients (attempted) intubated

• Fraction = unanticipated difficult intubations [False Negative]/true difficult intubations ([False negative] + [True Positive])

• Sensitivity

• Specificity

• Positive predictive value

• Negative predictive value

• Positive Likelihood Ratio = (Sensitivity/(1-Specificity))

• Negative Likelihood Ratio = ((1-Sensitivity)/Specificity)

• The Receiver Operating Characteristic curve. A graphical representation of sensitivity as a function of (1-Specificity). Applicable for comparison of predictive models.

An analogue outcome measurement will be done for mask ventilation.

### The simplified airway risk index (SARI)

The SARI model consists of seven predictors for difficult direct laryngoscopy:

1. Mouth opening

2. Thyromental distance

3. Mallampati class

4. Neck movement

5. Ability to prognath

6. Weight

7. History of difficult intubation

**Figure 2:**
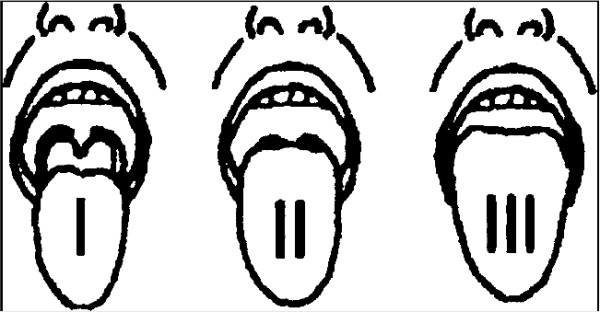
Original Mallampati grades.

**Figure 3:**
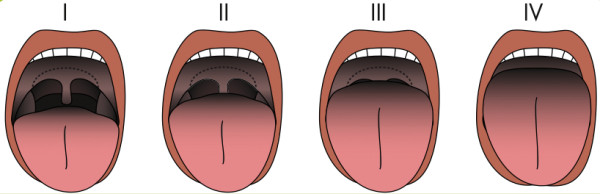
Modified Mallampati classification.

The SARI uses the original Mallampati grade, whereas for data entry in DAD a modified Mallampati class [20] will be used (Figure 2 and Figure 3). The Mallampati grades contribute to the SARI score as follows: Grade III → 2 points; Grade II → 1 point; Grade I → 0 points.

**Figure 4:**
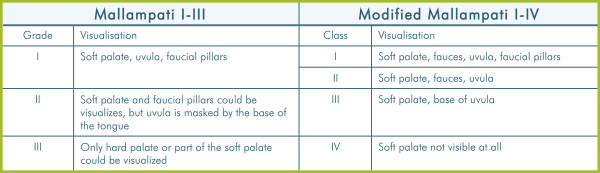
**Mallampati comparison.** The original Mallampati uses three grades of visualisation and the modified Mallampati uses four classes.

The original Mallampati grade I approximately corresponds to the modified Mallampati classes I and II, the original Mallampati grade II approximately corresponds to the modified Mallampati class III, and the original Mallampati grade III corresponds to the modified Mallampati class IV (Figure 4).

### Predictors of difficult and impossible mask ventilation

The following parameters that correlate to difficult/impossible mask ventilation are registered in the SARI group:

1. Changes in the neck due to radiation

2. Presence of beard

3. BMI ≥30 kg/m^2^

4. Age ≥57 years

5. Modified Mallampati score III or IV

6. Severely limited jaw protrusion

7. Snoring

8. Sleep apnoea

The predictors for DMV are already recorded in the DAD and the SARI except for the four listed below. Consequently, departments allocated to the SARI group also record:

1. Changes in the neck due to radiation

2. Presence of beard

3. Snoring

4. Sleep apnoea

### Definition of difficult intubation and difficult mask ventilation

**Figure 5:**
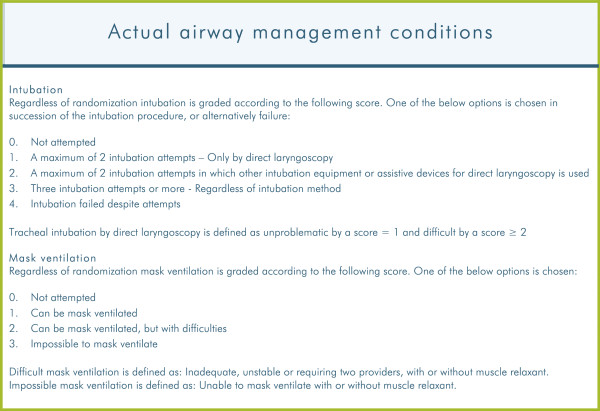
**Actual airway management conditions.** Intubation and mask ventilation score in the Danish Anaesthesia Database.

El-Ganzouri et al. classified laryngoscopy view after Cormack and Lehane’s Class I to IV grading system [21] and used it as a surrogate measure for difficult intubation. In the DIFFICAIR trial, an intubation score is programmed in the DAD based on numbers of attempts and use of equipment (Figure 5). Thus describing the actual circumstances regarding the intubation. An equivalent score is programmed for mask ventilation.

### Data registration on the anaesthesia record

The variables in the SARI model are recorded on an appendix to the anaesthesia record either on a pre-printed label adhered to the record form or on a pre-printed supplementary form that is stapled on to the anaesthesia record.

### Data registration in the DAD

**Figure 6:**
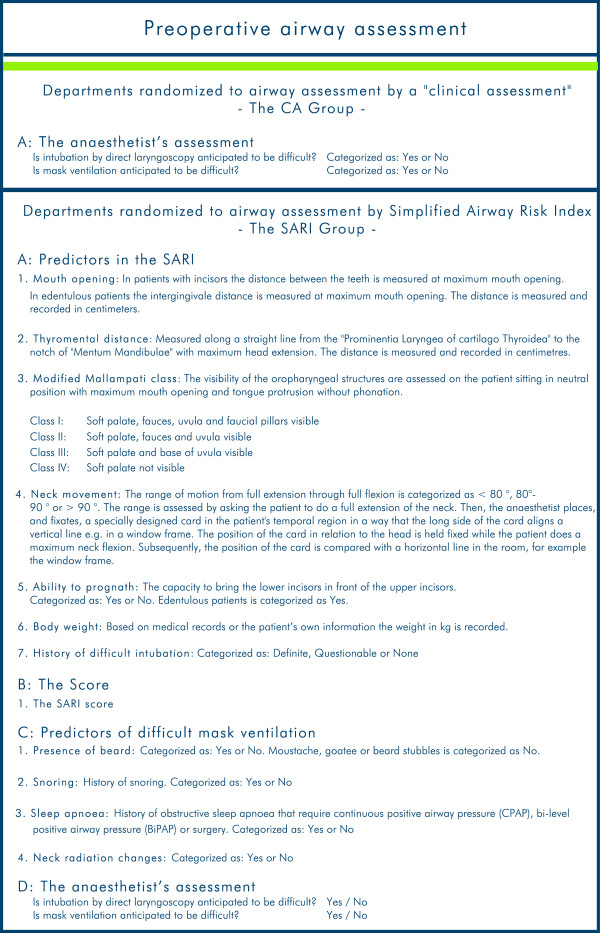
**Preoperative airway assessment.** Registration of the preoperative airway assessment in the Danish Anaesthesia Database is dependent on the randomization and group allocation.

**Figure 7:**
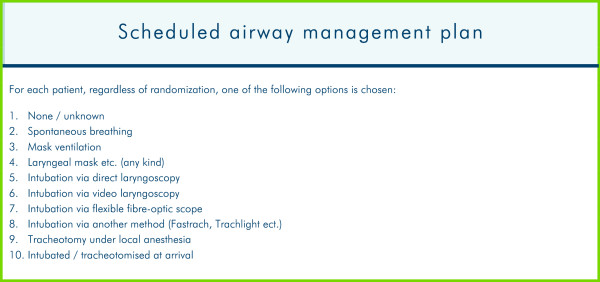
**Scheduled airway management.** The scheduled airway management plan entered into the Danish Anaesthesia Database.

For all patients the following variables are recorded: i) Preoperative airway assessment (Figure 6); ii) Scheduled airway management (Figure 7); iii) Actual airway management (Figure 5).

The registration of the preoperative airway assessment differs according to group.

SARI group:

A. Predictors included in the SARI model

B. The SARI score

C. Predictors of difficult mask ventilation

D. The anaesthetist’s assessment:

Is intubation by direct laryngoscopy anticipated to be difficult? Yes/No.

Is mask ventilation anticipated to be difficult? Yes/No.

CA group:

The CA group uses variables that are already registered in the DAD.

A. The anaesthetist’s assessment:

Is intubation by direct laryngoscopy anticipated to be difficult? Yes/No.

Is mask ventilation anticipated to be difficult? Yes/No.

The seven predictors of difficult intubation contained in the SARI model are registered in the database. Based on these data, the DAD auto-generates a SARI score. In addition, the values of the four predictors of DMV are registered. Despite knowing the SARI score at the preoperative assessment, the anaesthetist’s assessment of anticipated difficulties can differ from the SARI score. Hence, the score is only meant to be indicative of intubation difficulties or not. Following airway management, the actual airway management conditions are finally recorded (Figure 5).

### Estimation of sample size

The required number of patients for the detection or rejection of a given effect of the intervention in a cluster randomized trial is calculated by adjusting the required number of patients in a corresponding individually randomized trial with the degree of variation between the clusters (between-cluster variance) [22]. This method is analogue to adjustment with the intra-cluster correlation coefficient [22]. Deviations from the individual sample size estimation are necessary [18] and the calculation can be based on comparison between the groups at cluster level, if the following four conditions are met: i) the intervention is used strictly on cluster level; ii) patients and anaesthetists (intubators) do not migrate between clusters; iii) patients/anaesthetists (intubators) cannot be selected for, or by themselves select/deselect, the intervention; iv) all patients in each cluster are exposed to the intervention and no patient chooses a cluster based on preference for one type of airway assessment.

The estimated number of departments required for inclusion in the trial is based on data extraction from the DAD on patients who had unanticipated difficult tracheal intubations.

There were no previous records of the trial’s primary outcome measure, “unanticipated difficult intubation”. A baseline study was conducted using data from the DAD generated between 1 January and 1 June 2011. A total of 29 departments met the requirements of cluster size and registration of unanticipated difficult intubations. There were a total of 31,268 intubations or intubation attempts, of which 584 were unanticipated difficult, corresponding to a proportion of 1.87%. We calculated the cluster size and proportions of unanticipated difficult intubation for each department and used this to calculate the “between-cluster variance”.

The sample size estimation was further adjusted for the stratification of departments according to their proportions of unanticipated difficult intubation. The estimation was also adjusted according to sample size adjustments in matched cluster trials. We assume that the coefficient of variation, k, is similar in both the CA and SARI groups. Thus, the sample size estimation based on the baseline study data led to k = 0.25, corresponding to an intra-cluster correlation coefficient of 0.002, and an adjusted average cluster size of 1,611 patients.

In the stratified randomization, choosing a power of 80% (power = 1-β, with β being the maximal risk of type 2 error) and a maximal risk of type 1 error of 5% (α = 0.05, two-sided), we intend to be able to detect or reject a 30% relative risk reduction from 1.87% to 1.31%. Given these assumptions, approximately 30 departments are required for the trial: 15 in the SARI group and 15 in the CA group. In this case, it is possible to show that number needed to treat (NNT) is 180 or less. Therefore, we will be able to avoid one unanticipated difficult intubation for every 180 airways assessed by the SARI score instead of a non-specified preoperative airway assessment, if the trial detects a statistically significant difference.

The trial period will be 15 months. We have included 28 departments, randomized and stratified by a proportion of unanticipated difficult intubation less or greater than 2%. The departments are expected to have an average cluster size of approximately 2,500 patients, equalling allocation of approximately 35,000 patients for each trial group.

### Statistical analysis

The observed risk factors provide the basis for calculating the SARI score and for preoperative anticipation of a difficult intubation or not. Comparisons between the outcomes of the trial groups will be done on an individual level according to our sample size estimation. In the primary adjusted analysis, the number of patients having an unanticipated difficult (easy) intubation will be compared between the two trial groups with a logistic regression analysis adjusted for stratification variable of baseline proportions of unanticipated difficult (easy) intubation and clustering [23]. The OR for unanticipated difficult (easy) intubation comparing the SARI group with the CA group, and its 95% confidence limits, will be estimated [23]. In an unadjusted analysis, the number of patients having an unanticipated difficult (easy) intubation will be compared between the two trial groups with a χ^2^ test. The difference in proportions of unanticipated difficult (easy) intubation will be given with 95% confidence limits. Finally, an adjusted analysis using both stratification variables, the clustering, elective/acute, sex, age, use of neuromuscular blocking agents, and BMI will be performed [7,24].

The accuracy of the SARI score will be compared with the accuracy of the clinical assessment in the CA group on predicting difficult intubation. Additionally, the clinical assessment of the CA group will be compared with the clinical assessment of the SARI group based on the SARI score. That is, anticipations of intubation difficulties based on a clinical assessment only versus anticipations of intubation difficulties based on a clinical assessment while knowing the SARI score. In all analyses, a *P* value less than 0.05 will be considered statistically significant.

### Implementing and sustaining the experimental intervention

Before initiation of the trial, anaesthetists, in departments randomized for the use of the SARI model, were systematically trained in the performance of proper airway assessment according to the SARI score. This ensures uniform and high quality airway assessments [25]. A tutorial film, describing the trial and the preoperative SARI airway assessment in detail, has been produced and was shown to all anaesthetists. In each department, a principal investigator was appointed to ensure individual training of anaesthesiologists in correct airway assessment at trial start and again after 6 months. All new employees also receive this training. A short description of the DIFFICAIR trial is included in the introduction material for new physicians and nurses on intervention departments. Posters were placed and flyers made available describing the SARI model. A card that fits uniform pockets was produced. The card includes a ruler and a protractor to facilite airway assessment.

A website, http://www.difficair.com, containing all information including the tutorial film, PowerPoint presentations and other tools for education, was programmed. Different material is available on the website for the SARI- and the CA group. Different access is granted via different passwords.

### Implementing and sustaining the control intervention

For the departments randomized to the CA group, there will be no changes in registration of data in the DAD compared to usual standards. On the anaesthesia record (or directly into the DAD), the preoperative airway assessment and the scheduled airway management are recorded before the anaesthesia and actual airway management begins.

For both groups, a mask ventilation and/or an intubation score (Figure 5) is registered on the anaesthesia record or directly in the DAD during anaesthesia and immediately after airway management.

Regardless of trial group, the DAD registration is performed during or immediately after the end of anaesthesia. Data is entered via a computer workstation with an Internet connection to DAD. The anaesthetist who performed the airway management or the anaesthetist who completed the anaesthesia performs the registration.

In case of technical problems with the DAD, e.g., loss of Internet connection, a paper form corresponding to the electronic interface in DAD is used. Relevant personnel, e.g., a secretary, subsequently enter data from the form into the DAD at restoration of Internet connection.

After trial completion, data will be retrieved from the DAD, guarantying patients’ anonymity, following the rules of the Danish data protection agency. Anonymous data will be made accessible by other researchers through the Danish Data Archive.

### Data monitoring

Through the trial period, the degree of data completeness will be continuously monitored for each department. In case of a declining percentage of registration the principal investigator in the corresponding department will be contacted in order to restore the registration rate. The investigator group is blinded for all outcome measures during the trial period.

### Handling of incomplete data

Missing data exceeding a rate of 5% and with a statistical significant Little’s test, precluding analyses on the data set of complete cases, will be handled statistically through multiple imputation [26-28].

### Trial registration and ethics

The trial is a database research project involving registration of variables that are already being observed in the involved departments to varying degrees. The trial is without risks, side effects or inconvenience for the patient, and the trial protocol includes no specific dictation on airway management.

The Scientific Ethics Committee of Copenhagen County consents that the protocol should not be reported to the committee system (Journal No.: H-3-2012-FSP2). Individual informed consent from the patients is not necessary, which is essential for the feasibility. However, informed consent from every participating department by the Head of Department was acquired. The trial is approved by The Danish Data Protection Agency (j.nr.: 2007-58-0015/HIH-2011-10, I-Suite nr: 02079) and is registered at http://www.clinicaltrials.gov (NCT01718561).

### Publications

The protocol is written according to the SPIRIT 2013 recommendations [29]. Results of the trial will be reported according to the CONSORT statement for cluster randomized trials [18] and the STROBE criteria [30].

Manuscripts are written for publication in international peer reviewed journals. First author is Anders K. Nørskov, MD, Department of Anaesthesiology, Nordsjællands Hospital – Hillerød. Additional authors are Jørn Wetterslev, Chief Physician, MD, PhD, Copenhagen Trial Unit, Rigshospitalet; Charlotte V. Rosenstock, Consultant, MD, PhD and Lars H. Lundstrøm, MD, PhD, both from the Department of Anaesthesiology, Nordsjællands Hospital – Hillerød. The DIFFICAIR steering committee will grant additional authorship in accordance with the Vancouver rules and all trial site investigators are acknowledged with co-authorships.

Based on the trial results and international literature we hope to contribute to a national recommendation for preoperative airway assessment and its subsequent implementation.

The method used in the study is “state of the art” for testing an implementation of a recommendation [31,32]. All manuscripts will be submitted for publication to international peer-reviewed journals, published in annual reports from the DAD, and presented at national and international congresses.

Side studies will be allowed in accordance with the steering committee.

### Timeline

2011: Applications for funding. Acceptance from ethical committee. Baseline study and sample size calculation.

2012: Applications for funding. Written consent to randomization from 28 departments of anaesthesia. Revision and programming of the DAD. Education of intervention departments.

End 2012: First patient inclusion.

End 2013: Last patient inclusion.

Early 2014: Data analysis. Writing and submission of main manuscripts for publication.

### Collaborations and finances

The trial is done in collaboration between the Danish Anaesthesia Database; Department of Anaesthesiology, Copenhagen University Hospital, Nordsjællands Hospital – Hillerød; Copenhagen Trial Unit, Centre for Clinical Intervention Research, Copenhagen University Hospital, Rigshospitalet; and 28 Danish departments of anaesthesia. All participating departments provided written consent for inclusion and randomization for the trial. The DAD steering committee supports the DIFFICAIR trial and the data extraction is done in agreement with the committee.

The investigators have no financial ties to private companies or foundations and no potentially conflicting interests in the project.

The study is fully funded by the Tryg Foundation; the Research foundation at Copenhagen University Hospital, Nordsjællands Hospital – Hillerød; DASAIMs fund; and resources at local trial sites. None of the funding sources has any influence on protocols, data handling or publications.

### Discussion and perspective

It is innovative to use a national clinical database as the basis for a randomized clinical trial. The method can serve as a precedent for implementation of evidence-based recommendations and database registrations.

The trial will forward understanding of how to predict and reduce the unanticipated difficult intubation and mask ventilation and how to produce evidence-based recommendations for airway assessment nationally and internationally.

## Trial status

The trial was initiated on October 1, 2012 through DAD recording in all intervention and control departments. Two control departments still await connection to the DAD registry. Nevertheless, they are expected to meet the minimum inclusion criteria before trial termination.

Patient recruitment was ongoing at time of submission of the manuscript.

The trial ends at the end of 2013.

## Abbreviations

ASA: American Society of Anesthesiologists; BMI: Body mass index; CA: Clinical assessment; DAD: Danish Anaesthesia Database; DMV: Difficult mask ventilation; NNT: Number needed to treat; SARI: Simplified Airway Risk Index.

## Competing interests

The authors declare that they have no competing interests.

## Authors’ contributions

AN participated in the study design, contributed to the use of study methodology, carried out the statistical calculations and drafted the manuscript. CR participated in the design of the study and helped to draft the manuscript. JW participated in the design of the study, contributed to the use of study methodology and statistical calculations, and helped to draft the manuscript. LL participated in the design of the study and the use of study methodology, and helped with the statistical calculations and the drafting of the manuscript. All authors read and approved the final manuscript.
